# AHDC1/Gibbin: a master regular of chromatin structure and gene transcription

**DOI:** 10.3389/fneur.2024.1388029

**Published:** 2025-01-06

**Authors:** Linyi Li, Chao Zhang, Yanwen Qin

**Affiliations:** ^1^Beijing Anzhen Hospital, Capital Medical University, Beijing Institute of Heart Lung and Blood Vessel Disease, Beijing, China; ^2^Fundamental Research Center, Shanghai Yangzhi Rehabilitation Hospital (Shanghai Sunshine Rehabilitation Center), Shanghai, China

**Keywords:** AHDC1, Gibbin, Xia-Gibbs syndrome, chromatin, DNA methylation, gene transcription

## Introduction

AT hook DNA-binding protein (AHDC1/Gibbin) is a nuclear protein with currently undefined function and structure. *De novo* nonsense or frameshift mutations in the *AHDC1* gene have been identified as the causative factor for Xia-Gibbs syndrome (XGS, OMIM#615829) in 2014, a neurodevelopmental disorder characterized by intellectual disability and developmental delay (1). XGS occurs in infancy and is often accompanied by hypotonia, followed by global developmental and expressive language delays, ultimately leading to intellectual disabilities (2). Whole-exome sequencing has identified more than 390 individuals worldwide with XGS, with the number continually increasing as sequencing diagnostic techniques are widely adopted. The XGS registry was established in 2014, with detailed clinical records contributed by over 100 families who have consented to participate in additional research activities (3), presenting the opportunity to uncover the mutation spectrum and pathogenesis of XGS. Given the clinical importance, urgent investigations are required to explore the functional role of AHDC1/Gibbin and unravel the molecular mechanisms underlying its pathogenic mutations. In this opinion article, we synthesize recent findings on the pathogenic variants of the *AHDC1* gene and its protein biological functions, highlighting several critical scientific inquiries that demand immediate attention.

## The pathogenesis of XGS remains elusive

**Figure 1:**
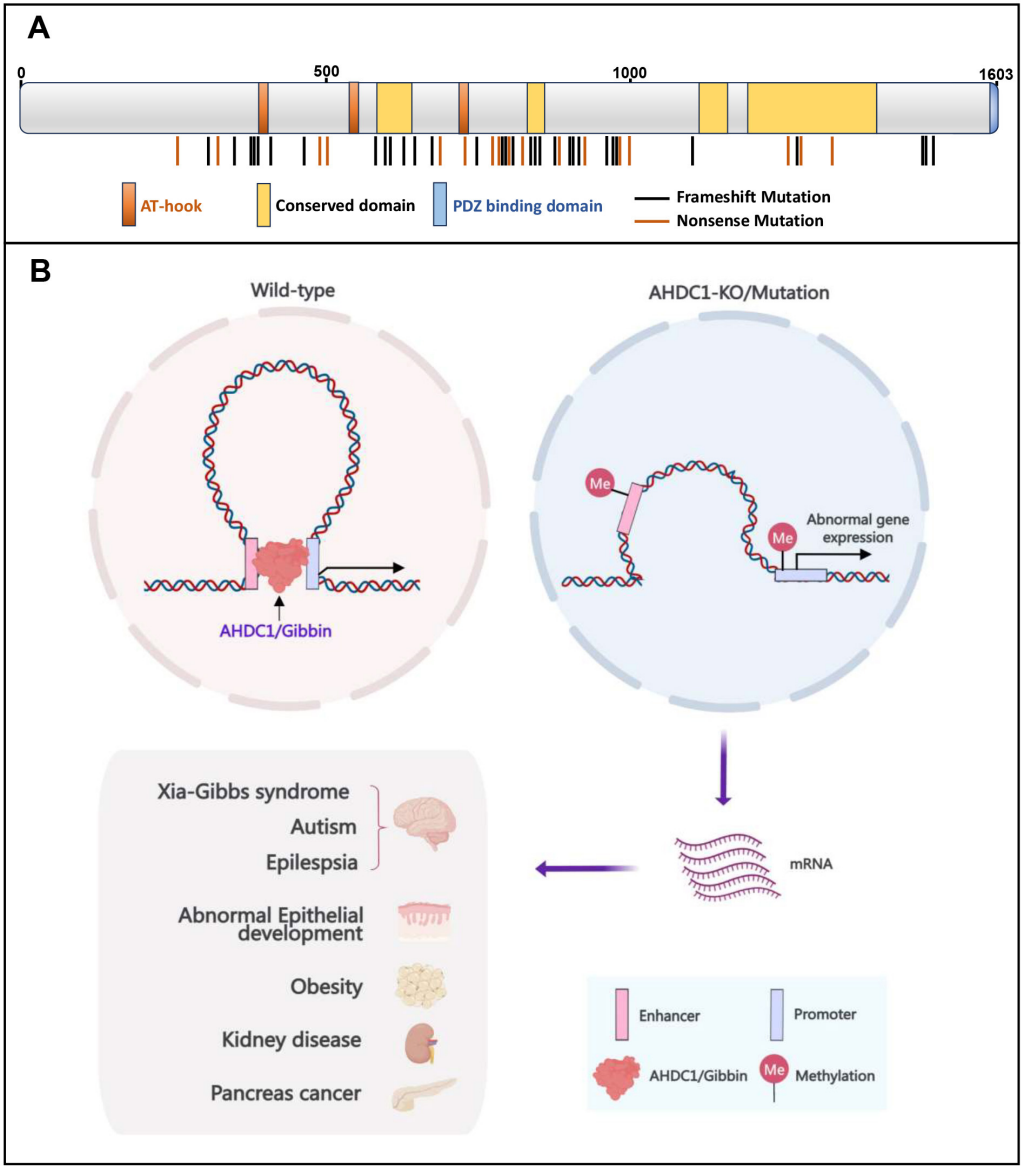
Graphic abstract of AHDC1/Gibbin. **(A)** AHDC1/Gibbin is a DNA-binding protein that contains AT hooks; however, the identification of its critical functional domains is still pending. The vertical lines depicted below the schematic indicate the mutation positions associated with Xia-Gibbs syndrome. **(B)** Schematic depiction of the multifaceted role of AHDC1/Gibbin in regulating DNA methylation, maintaining promoter-enhancer chromatin contacts, modulating gene expression, and its potential implication in diverse diseases.

XGS primarily arises from spontaneous pathogenic truncating mutations of the *AHDC1* gene on chromosome 1 at locus 1p36.11. Notably, only the sixth exon of the *AHDC1* gene encodes protein, while the rest are non-coding regions. The coding sequence exhibits remarkable conservation among species at the nucleotide level. Similarly, the 3′ non-coding exons maintain a level of conservation akin to that of coding exons, implying their potential importance (1). To date, pathogenic mutations have been discovered in most parts of the *AHDC1* gene, predominantly as frameshift mutations (4) (Figure 1A). However, the exact correlation between these *AHDC1* mutation sites and the clinical phenotypes of patients remains incompletely understood. Clinical studies involving 20 individuals carrying 16 different *AHDC1* mutations in XGS have suggested a potential association between truncating mutations at the protein's C-terminus and non-verbal disabilities (3). However, it is worth noting that the same recurrent mutations observed in multiple patients exhibit diverse phenotypic variations among different individuals (3). Furthermore, a nonsense mutation at the N-terminus of the AHDC1/Gibbin protein results in a shorter form of the mutant protein, which demonstrates more severe phenotypic effects compared to proteins with C-terminal truncations (5). These observations underscore the phenotypic heterogeneity caused by *AHDC1* mutations, which has been well-summarized by Khayat et al. (4).

The mechanisms underlying how *AHDC1* mutations result in the XGS phenotype are unclear. Studies suggest that *AHDC*1 mutations lead to varying molecular consequences, possibly through dominant negative effects, gain of function (GoF), or haploinsufficiency. Chander et al. observed that truncating pathogenic mutations of *AHDC1* can escape nonsense-mediated mRNA decay to produce truncated versions, while individuals with large deletions in the *AHDC1* gene display almost normal expression levels, hinting at possible compensatory mechanisms (2). This supports the theories of dominant negative or GoF mechanisms linked to *AHDC1* variations. Missense mutations in *AHDC1* are also likely to produce full-length proteins that could act through similar mechanisms (6, 7). Additionally, large *de novo* deletions encompassing the entire *AHDC1* locus have been associated with XGS (8–10). Disruptions in the 5′UTR region of *AHDC1* leading to reduced gene expression have also been documented (11), supporting the hypothesis that *AHDC1* haploinsufficiency may contribute to its related developmental disorders. More research is essential to understand the effects of different mutations on *AHDC1* expression and the protein's structure and function.

## The functional study of AHDC1/Gibbin is urgent and challenging

Investigating the functional loss or gain due to *AHDC1* mutations is crucial for understanding XGS pathogenesis and may aid in therapeutic development and inform research on other developmental disorders. However, correlating specific mutation sites to phenotypic outcomes remains a challenge due to limited understanding and statistical data. Deciphering these complexities will depend on a comprehensive understanding of the full range of functions of the AHDC1/Gibbin protein and a detailed examination of how various mutations affect its activity.

The study of AHDC1/Gibbin is fraught with difficulties: the large, 1603-amino-acid protein complicates the understanding of its functional domains, purification of recombinant proteins, crystal structure analysis, and *in vitro* functional studies. The cellular state of AHDC1/Gibbin (monomeric or multimeric), its nuclear distribution patterns (4), and its interacting partners remain enigmatic, presenting barriers to delineating its role in diseases. Furthermore, a lack of animal models for direct observation and the gene's ubiquitous expression across tissues pose additional challenges in unraveling AHDC1/Gibbin's function. These complexities make it difficult to advance our understanding of AHDC1/Gibbin's role in XGS and beyond.

## Is AHDC1/Gibbin a chromatin structure and gene transcription regulatory factor?

Recently, Collier et al. (12) discovered that AHDC1/Gibbin serves as a crucial controller in mesodermal lineage specification, thus providing a potential framework for understanding the pathogenesis of XGS. Through its interaction with chromatin regulatory factors and DNA methylation, AHDC1/Gibbin plays a pivotal role in preserving accurate chromatin contacts between promoters and enhancers of mesodermal genes during the differentiation process. Consequently, the loss of AHDC1/Gibbin disrupts these chromatin contacts, resulting in the dysregulated expression of mesodermal-specific genes and the consequent abnormal development of the epithelial tissue. However, the mechanism through which AHDC1/Gibbin contributes to chromatin looping has yet to be fully elucidated. Nevertheless, this study is highly commendable as it uncovers a comprehensive molecular landscape of AHDC1/Gibbin during the differentiation of hESC into epithelial lineages.

Although this study does not definitively characterize the precise function of AHDC1/Gibbin, it suggests that AHDC1/Gibbin is a crucial player in chromatin structural organization and transcription regulation. It is implicated in the modulation of DNA methylation and chromatin contacts, similar to other chromatin organization regulators such as methyl-CpG binding protein 2 (MECP2) and activity dependent neuroprotective protein (ADNP). However, the specific molecular mechanisms underlying AHDC1/Gibbin's impact on DNA methylation and its interaction with chromatin are still unknown. Furthermore, AHDC1/Gibbin's transcription regulatory and lineage-determining roles are limited to cellular differentiation, similar to MECP2 and ADNP. The interacting protein profiles of AHDC1/Gibbin also resemble those of MECP2 and ADNP. Additionally, the variation in protein localization patterns of AHDC1/Gibbin in the cell nucleus due to different mutations (4) suggests that structural changes in AHDC1/Gibbin protein contribute to XGS pathogenesis, mirroring potential functions similar to MECP2 (13). Therefore, it is reasonable to hypothesize that AHDC1/Gibbin dysfunction could affect specific genes through physical factors in three-dimensional chromatin organization, similar to the role of MECP2 and ADNP. Phenotypic heterogeneity caused by *AHDC1* mutations may also potentially stem from differences in chromatin organization and transcriptional expression patterns arising from distinct *AHDC1* mutation sites. Future studies might validate this hypothesis by investigating the three-dimensional structure of normal and mutated AHDC1/Gibbin proteins and their impact on chromatin organization.

## AHDC1/Gibbin's potential involvement in multiple diseases–beyond Xia-Gibbs syndrome

Changes in chromatin structure and DNA methylation have been shown to contribute to the development and progression of various neurological and metabolic disorders, such as epilepsy, Alzheimer's disease, schizophrenia, autism, cancer and obesity. Considering that the AHDC1/Gibbin protein may serve as a regulator of chromatin structure, DNA methylation, and gene transcription, structural and functional deficits in this protein are likely to induce widespread disruptions in gene expression. Support for these notions has been obtained from the work by Collier et al. (12), which, when coupled with the globally consistent gene expression pattern of *AHDC1* observed across tissues, hints at the potentially extensive pathological reach of AHDC1/Gibbin dysfunction. Current research also supports that AHDC1/Gibbin plays a multifaceted and diverse role. For example, *AHDC1* gene mutations have been discovered in patients with syndromic obesity (14). We recently discovered that mice with *Ahdc1* deficiency exhibit notable obesity and energy metabolism disruption (15). Additionally, Xia-Gibbs syndrome patients experience both epilepsy and autism (3); whole-genome sequencing has revealed rare *AHDC1* germline variants in first-degree relatives of familial pancreatic cancer patients (16); and there is a heightened risk of long-term kidney disease in patients with rare missense variants in *AHDC1* (17). Further investigations are imperative to achieve a comprehensive comprehension of AHDC1/Gibbin's involvement in these diverse diseases and to illuminate the complete range of pathological consequences resulting from *AHDC1* mutations.

Despite the successful generation of an *Ahdc1* knockout mouse model by Collier et al. (12), both homozygous and heterozygous mice were unable to survive. Considering that XGS is typically diagnosed after birth and patients can live into adulthood, the availability of a viable animal model is essential for accurately mimicking the natural history of the disease and investigating potential therapeutic approaches. We have established a mouse model with a heterozygous deletion targeting exon 6 of *Ahdc1* and found a ~50% reduction in *Ahdc1* gene expression (15), using a different strain and breeding method than the Collier's study. While Collier et al. used the C57BL/6J strain for mosaic CRISPR mutants (12), we opted for the C57BL/6N strain and successfully generated heterozygotes through multiple rounds of *in vitro* fertilization. Our research suggests that this model shows neurobehavioral abnormalities and can partially mimic the phenotype of XGS patients (unpublished). Future studies should focus on creating mouse models with pathogenic mutations and investigating the tissue-specific and adult biological functions of AHDC1/Gibbin through conditional knockouts.

## Concluding remarks

In summary, unraveling the molecular mechanism of XGS linked to *AHDC1* mutations requires future investigation into AHDC1/Gibbin's structure and function and its mutation-induced dysfunctions using advanced technologies at *in vitro*, cellular, and animal levels. The disruption of AHDC1/Gibbin protein's role in chromatin structure, DNA methylation, and gene regulation could be a key mechanism underlying XGS (Figure 1B). Understanding AHDC1/Gibbin's structure and biological functions, as well as the determinants and mechanisms governing its regulation of DNA methylation and chromatin organization, will provide valuable insights into chromatin regulatory mechanisms and aid in the development of therapeutic interventions for Xia-Gibbs syndrome and related diseases.
